# Ecological patterns and processes of temporal turnover within lung infection microbiota

**DOI:** 10.1186/s40168-024-01780-6

**Published:** 2024-03-25

**Authors:** Helen Gavillet, Lauren Hatfield, Andrew Jones, Anirban Maitra, Alexander Horsley, Damian Rivett, Christopher van der Gast

**Affiliations:** 1 Department of Applied Sciences, Northumbria University, Newcastle, UK; 2 Department of Life Sciences, Manchester Metropolitan University, Manchester, UK; 3 Manchester Adult Cystic Fibrosis Centre, Manchester University NHS Foundation Trust, Manchester, UK; 4 Royal Manchester Children’s Hospital, Manchester University NHS Foundation Trust, Manchester, UK; 5 Division of Infection, Immunity and Respiratory Medicine, University of Manchester, Manchester, UK; 6 Department of Natural Sciences, Manchester Metropolitan University, Manchester, UK; 7 Department of Respiratory Medicine, Northern Care Alliance NHS Foundation Trust, Salford, UK

**Keywords:** Cystic fibrosis, Lung microbiome, Respiratory microbiome, Chronic infection, Temporal dynamics, Species-time relationships, Island biogeography, Ecological patterns and processes, Lung ecology, Microbiome ecology

## Abstract

**Background:**

Chronic infection and consequent airway inflammation are the leading causes of morbidity and early mortality for people living with cystic fibrosis (CF). However, lower airway infections across a range of chronic respiratory diseases, including in CF, do not follow classical ‘one microbe, one disease’ concepts of infection pathogenesis. Instead, they are comprised of diverse and temporally dynamic lung infection microbiota. Consequently, temporal dynamics need to be considered when attempting to associate lung microbiota with changes in disease status. Set within an island biogeography framework, we aimed to determine the ecological patterns and processes of temporal turnover within the lung microbiota of 30 paediatric and adult CF patients prospectively sampled over a 3-year period. Moreover, we aimed to ascertain the contributions of constituent chronic and intermittent colonizers on turnover within the wider microbiota.

**Results:**

The lung microbiota within individual patients was partitioned into constituent chronic and intermittent colonizing groups using the Leeds criteria and visualised with persistence-abundance relationships. This revealed bacteria chronically infecting a patient were both persistent and common through time, whereas intermittently infecting taxa were infrequent and rare; respectively representing the resident and transient portions of the wider microbiota. It also indicated that the extent of chronic colonization was far greater than could be appreciated with microbiological culture alone. Using species-time relationships to measure temporal turnover and Vellend’s rationalized ecological processes demonstrated turnover in the resident chronic infecting groups was conserved and underpinned principally by the deterministic process of homogenizing dispersal. Conversely, intermittent colonizing groups, representing newly arrived immigrants and transient species, drove turnover in the wider microbiota and were predominately underpinned by the stochastic process of drift. For adult patients, homogenizing dispersal and drift were found to be significantly associated with lung function. Where a greater frequency of homogenizing dispersal was observed with worsening lung function and conversely drift increased with better lung function.

**Conclusions:**

Our work provides a novel ecological framework for understanding the temporal dynamics of polymicrobial infection in CF that has translational potential to guide and improve therapeutic targeting of lung microbiota in CF and across a range of chronic airway diseases.

Video Abstract

**Supplementary Information:**

The online version contains supplementary material available at 10.1186/s40168-024-01780-6.

## Background

For people living with cystic fibrosis (CF) the primary cause of morbidity and early mortality is chronic lung infection and consequent inflammation [1]. Regular microbiological surveillance of airway secretions throughout the life of a CF patient is considered best practice and is central to clinical care in CF [2]. It is used to distinguish intermittent or chronic infection status of CF canonical pathogens, guide the choice of antimicrobial therapy, indicate the effectiveness of interventions against current chronic infection, and enable timely treatment of new infection to prevent the establishment of chronic infection with its association with poor long-term outcomes [2–5]. Following diagnosis of acute pulmonary exacerbation, increased surveillance for the duration of treatment is also recommended to direct and assess efficacy of antibiotic therapy [2]. Moreover, surveillance is used to categorize CF patients by their lead chronic CF pathogen, dictating which segregated outpatient clinics they attend to prevent cross-infection between patients infected with, for example, *Pseudomonas aeruginosa*, methicillin-resistant *Staphylococcus aureus*, or *Burkholderia cepacia* complex members [6].

Clinical microbiological surveillance in CF is predominantly culture-based and is driven by ‘one microbe, one disease’ concepts of infection pathogenesis originating from Koch’s postulates [7, 8]. Where culture is used to report the presence or absence (and not abundance) of targeted CF pathogens [3, 4]. Conversely, basic research using molecular-based approaches developed for microbial ecology has established that lung infection in CF is unquestionably polymicrobial, involving a complex and interacting lung microbiome, e.g. [7–12]. More broadly, it is the case that the lower airways in health and across a range of respiratory diseases contain diverse and dynamic microbiota, exemplifying the inadequacy of traditional models of lung microbiology and infection pathogenesis [13–15]. Nevertheless, therapeutic targeting of the lung microbiome translated into a clinical context, including in CF, remains challenging [7, 16].

Setting microbiome research within a theoretical ecology framework has long been promoted as a parsimonious and pragmatic solution to understanding and predicting the ecology of microbiota, regardless of habitat or system [17]. The theory of island biogeography specifically has been promoted as a useful framework to apply and adapt to respiratory microbiome ecology, as lungs can be considered as dynamic island habitats that are subject to species immigration and extinction through time [13, 18, 19]. Island biogeography is appealing in this context as it has temporal turnover, defined as “the number of species eliminated and replaced per unit of time”, as its central underpinning concept [20]. Originally developed in traditional ecology to predict spatial and temporal patterns of animal and plant species richness on oceanic islands [19]. It has subsequently been applied and adapted for the study of microbiota across a diverse range of ‘island’ types, including water-filled tree-holes [21, 22], seawater mesocosms [23], engineering machine sump tanks [24], and wastewater treatment systems [25, 26]. Although aspects of the theory have been applied in a cross-sectional context to lung microbiota, e.g., [7, 14], to our knowledge it has not been applied to the lung microbiota of individual patients followed through time.

This could be important for understanding the dynamics of chronic and intermittent infecting bacteria within the wider lung microbiota of a patient through time. Indeed, it has been recommended that temporal dynamics need to be considered when attempting to connect changes in human microbiota to changes in health status [27]. Currently, there is no ‘gold standard’ clinical definition of chronic infection [28]. However, a commonly used definition is the Leeds criteria, originally developed to define chronic *P. aeruginosa* infection in CF patients [4]. In brief, patients are considered chronically infected when > 50% of the preceding 12 months respiratory samples are culture-positive for *P. aeruginosa* [4]. We recently used a modification of the Leeds criteria to assess the infection status of *P. aeruginosa* and *S. aureus* in paediatric and adult CF patients, highlighting a striking underestimation of chronic infection using clinical microbiological culture when compared to targeted molecular approaches [3]. In a lung microbiome context, using high-throughput targeted amplicon sequencing to define the whole lung microbiota, we hypothesize that CF patients will be chronically colonized, as defined by the Leeds criteria, to a much greater extent and with a larger number of bacterial species than expected by clinical microbiological culture alone [3, 7].

In the current study, respiratory samples from paediatric and adult CF patients were prospectively collected over a period of up to 3 years. Set within an ecological framework and using the cornerstone concept behind the theory of island biogeography [19], we aimed to determine the ecological patterns and processes underpinning the temporal turnover of chronic and intermittent colonizing bacterial taxa within the lung microbiota of individual participating patients. To achieve this, we sought to define a chronic and intermittent status for all bacterial taxa colonizing the airways of individual patients visualised using persistence-abundance relationships of the wider lung microbiota. Using species-time relationships [22, 25, 29], we assessed the temporal turnover of the chronic and intermittent taxa groups and how they contributed to patterns of turnover in the whole lung microbiota within individuals and across patients. We then ascertained which ecological processes underpinned the temporal turnover of bacterial taxa within the chronic and intermittent taxa groups and the wider lung microbiota [22]. Finally, we related those ecological patterns and processes to lung function. Specifically, we used forced expiratory volume in 1 s (FEV_1_), expressed as a normalized percent of the predicted value (%FEV_1_), as it is currently the single best clinical indicator of health for individuals living with CF [7, 30].

## Methods

### Study and patient sampling

Patients were recruited as part of a longitudinal observational study of adults and children with CF cared for at two different CF centres [31]. Adults were recruited from the Manchester Adult CF Centre (Wythenshawe Hospital), and children from the Royal Manchester Children’s Hospital. Patients were required to be at least 5 years old, with a FEV_1_ of > 50% predicted at study entry. Patients or parents/guardians provided written informed consent and children provided assent. This study was reviewed and approved by the NHS Research Ethics Committee North-West, Lancaster (Ref 14/NW/1195). For the current study, paediatric and adult patients from the wider study were excluded from further analyses if they had provided < 6 samples over the sampling duration and if the maximum duration of sampling was less ≤ 730 days, i.e., did not extend into the 3rd year of the overarching study.

The clinical characteristics of patients included in the current study are summarised in Table 1. Patients were assessed at their usual clinic appointments by their regular clinical team and included routine and emergency visits. Spirometry was performed by the usual clinical team. Normal ranges for spirometry were those from the Global Lung Initiative [30]. At study entry, 93% of adults (14/15) were on long-term azithromycin treatment, while one patient (patient 110) was receiving long-term colistin. Seventy-three percent of paediatric patients (11/15) were also on long-term azithromycin, the remaining four (201, 212, 216, and 228) were not prescribed any long-term antibiotics. Sputum or cough swab samples were taken at each clinic visit for diagnostic and molecular microbiology. All sputum samples were spontaneously expectorated. Molecular microbiology samples were transported to the lab within three hours and stored at -80 °C prior to DNA extraction and PCR [32, 33]. Sputum samples were mixed and weighed prior to splitting and storage.

**Table 1:** Clinical characteristics of individual (A) paediatric and (B) adult patients

	Age^b^	Sex	CFTR Genotype1	CFTR Genotype2	CF-related diabetes	Pancreatic insufficiency	%FEV_1_^c^	SD	Exacerbations^d^
(A)									
201^a^	12	Female	F508del	G551D	No	Insufficient	84.4	2.7	3
203	6	Female	F508del	F508del	No	Insufficient	100.2	10.7	1
212	9	Female	F508del	F508del	No	Insufficient	83.9	6.3	4
213	6	Male	F508del	F508del	No	Insufficient	82.3	15.7	3
216	9	Female	SN549N	SN549N	No	Insufficient	117.8	5.2	1
217	8	Male	F508del	G542X	No	Insufficient	100.4	2.7	0
218	11	Male	F508del	F508del	No	Insufficient	89.6	6.6	2
219	8	Male	F508del	R56OT	No	Insufficient	98.7	1.4	0
223	8	Male	F508del	S1235R	No	Sufficient	110.0	1.7	0
228	6	Male	F508del	R553X	No	Insufficient	99.8	6.2	0
233	15	Male	F508del	F508del	No	Insufficient	71.2	9.9	1
240	11	Female	F508del	F508del	No	Insufficient	60.6	6.3	1
242^a^	6	Female	F508del	G551D	No	Insufficient	74.7	8.6	2
245	6	Male	F508del	F508del	No	Insufficient	92.0	14.2	3
246	9	Female	3849 + 10kbC > T	3849 + 10kbC > T	No	Sufficient	91.0	9.1	2
(B)									
101	22	Male	R334W	R75X	No	Sufficient	64.2	2.1	7
103	22	Male	F508del	3849 + 10kbC > T	No	Sufficient	79.7	7.9	1
104	28	Male	3849 + 10kbC > T	3849 + 10kbC > T	No	Sufficient	77.9	7.5	2
106	19	Male	F508del	F508del	No	Insufficient	57.2	8.0	8
108	35	Male	F508del	F508del	No	Insufficient	60.7	1.5	2
110	28	Female	F508del	F508del	No	Insufficient	120.1	2.7	1
112	24	Male	F508del	F508del	No	Sufficient	93.2	4.5	1
113	21	Female	F508del	Delexon2-3	No	Insufficient	58.1	1.4	3
114	21	Male	F508del	F508del	No	Insufficient	87.1	1.9	0
116	24	Male	R117H	S549N	No	Sufficient	99.6	2.1	0
118	25	Female	F508del	F508del	No	Insufficient	73.8	4.2	3
119	20	Female	R553X	2622 + 1G- > A	Yes	Insufficient	47.9	2.9	3
120	19	Male	F508del	F508del	No	Insufficient	78.6	3.3	0
121	29	Male	F508del	R117H	No	Sufficient	92.9	3.7	1
140	17	Male	F508del	2622 + 1G > A	No	Sufficient	47.3	6.9	6

^a^Patients receiving Ivacaftor CF transmembrane conductance regulator (CFTR) modulator therapy

^b^Age in years at the start of the study

^c^Mean lung function ± standard deviation (SD) over the course of the study

^d^Number of acute pulmonary exacerbations experienced over the course of the study

### Sequencing

Nucleic acid extraction was performed on sputum and cough swab samples as previously described, with a modification for the latter sample type [34]. As an alternative to the wash stage for sputum, cough swabs were saturated in sterile phosphate buffer solution for 5 min, then squeezed with sterile tweezers to extract as much material as possible. The resulting solution was then introduced at the bead-beating stage and the protocol continued as normal for the sputum samples thereafter [3].

Following DNA extraction, approximately 2 ng of template DNA was amplified using Q5 high-fidelity DNA polymerase (New England Biolabs, Hitchin, UK) using a paired-end sequencing approach targeting the bacterial 16S rRNA gene region (V5–V6) as previously described [35]. Pooled barcoded amplicon libraries were sequenced on the Illumina MiSeq platform (V3 chemistry). Mock communities, DNA extract, and PCR negative controls were included in each sequencing run [35]. Sequence processing and analysis were carried out in R (Version 4.0.1), utilising the package DADA2, as previously described [36]. Raw sequence data have been deposited in the European Nucleotide Archive under study accession number PRJEB62148. All sequences were putatively assigned genus or species level identification by using the GTDB database [37] and then any remaining non-assigned ASVs were run through BLAST [38]. Given the length of the ribosomal sequences analysed, species identities should be considered putative. A sequence match of 97% or more when run through the databases was required for identification.

### Statistical analysis

All regression analyses, coefficients of determination (*R*^2^), degrees of freedom, *F*-statistics, and significance (*P*) were calculated using XLSTAT v2018.1 (Addinsoft, Paris, France). Kruskal–Wallis analyses.

in conjunction with the post hoc Dunn test, were performed in XLSTAT.

We used a temporal variation of the occupancy-abundance relationship, which we term the persistence-abundance relationship (PAR) to reflect the temporal nature of this study [11, 39]. Wherein, in place of plotting a measure of species occupancy of spatially separated sites or habitats against species abundance, temporal persistence was substituted for occupancy; defined here as the percentage number of samples each bacterial taxon was observed in the lung microbiota of a given patient over time.

Species-time relationships (STRs) were constructed using the moving window method [22]. New taxa were defined as the number of taxa present in the second/last sample of a window, but not observed in the first sample as a window was moved sequentially along a time series. Adjoining time sample points were taken pairwise moving along the time series, with the richness of the first sample added to the number of new taxa found in the second. For example, a 20-time point time series, richness in sample 1 is added to the new taxa observed in 2, then 2 and 3, 3 and 4, 4 and 5, etc., up to 19 and 20. The moving window approach incorporates multiple immigrations and extinctions of the same taxa through time, which would be anticipated in a time series of this study’s extent [22]. This is a key difference compared to other STR construction approaches, which only use the first appearance of each bacterial taxon, even though taxa can emerge and disappear multiple times across a time-series for a given microbiota [22]. All STRs were constructed and plotted in Microsoft Excel (Microsoft Corporation, Redmond, WA, USA).

To test to what extent temporal turnover within each patient’s lung microbiota was accounted for by Vellend’s rationalised ecological processes [40], patient microbiota were compared using a Monte Carlo procedure (1000 randomizations) to determine whether any two lung microbiota samples were more or less similar than expected by chance using the Raup and Crick probability-based index of similarity (*S*_RC_) [41]. For each patient, the ‘regional’ species pool was defined as all species that occurred through the time series for all patients. The *S*_RC_ probability-based index, which is independent of sample size and based on presence-absence data, was rescaled to range from 1 to − 1 [41], but, contrary to Chase et al., maintained as an intuitive measure of similarity and not dissimilarity [22]. Pairwise *S*_RC_ indices of ≥ 0.95 and ≤  − 0.95 are significantly similar or dissimilar, respectively, than expected by chance, and *S*_RC_ indices between 0.95 and -0.95 indicate similarity no greater than expected by chance [22, 41]. This has been extended to quantify which ecological processes shape differences between microbiota [22]. When *S*_RC_ is used as a similarity index, values near 1 (0.95 to 1) indicate homogenizing dispersal, values near − 1 (− 0.95 to − 1) indicate dispersal limitation, and values between 0.95 to − 0.95 indicate drift. *S*_RC_ indices were calculated using PAST v3.25 (www.nhm.uio.no/english/research/resources/past/) [22].

## Results

Here we analysed respiratory samples from 15 pediatric and 15 adult CF patients prospectively collected over a 3-year period. The clinical characteristics of individual patients are summarised in Table 1. The mean sampling duration ± standard deviation of the mean (SD) across the patients was 959.0 ± 120.8 days, with a minimum and maximum of 785 and 1166 days, respectively. The mean number of respiratory samples ± SD taken from participating patients was 8.7 ± 2.9, with a minimum and maximum of 6 and 20 samples, respectively. Following our previous work, a minimum of ≥ 6 samples was chosen, as less samples would have increased the likelihood of misclassifying chronic or intermittent infection status [3].

### Chronic and intermittent colonization

**Fig. 1:**
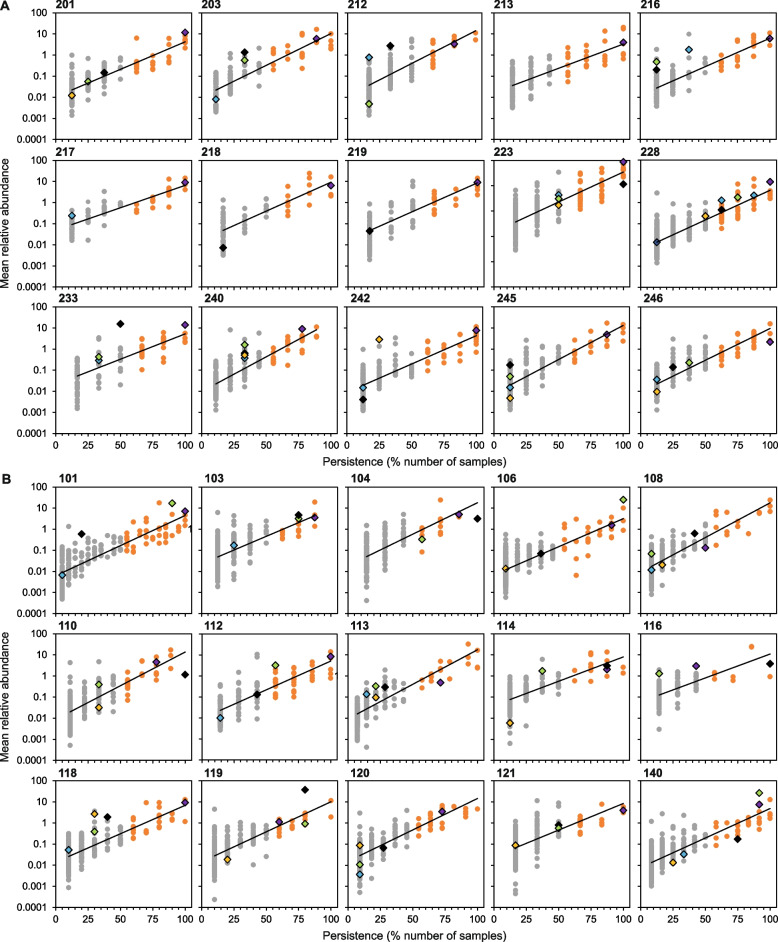
Persistence and abundance of chronic and intermittent colonizing bacteria within the lung microbiota of individual patients. Using a modification of the Leeds criteria, (**A**) paediatric and (**B**) adult patients were deemed to be chronically or intermittently colonized with a given bacteria if > 50% or ≤ 50% of samples, respectively, over the 3-year study period were positive by for that taxon. Chronic and intermittent colonizing taxa are denoted with orange and grey circles, respectively. When present, canonical pathogens are denoted with different coloured diamonds: *Pseudomonas aeruginosa*, black; *Staphylococcus aureus*, light green; *Stenotrophomonas maltophilia*, gold; *Burkholderia cepacia* complex members, light blue*; Haemophilus influenzae*, purple; and *Achromobacter xylosoxidans*, blue. Chronic or intermittent colonization status for all bacterial taxa within the microbiota of each patient is highlighted in the supplemental microbiota data (see Availability of data and materials). All persistence-abundance relationships were significant (*P* < 0.0001 in all instances). Regression statistics are provided in Supplementary Table S1

Using a modification of the Leeds criteria [3, 4], individual patients were considered to be chronically or intermittently colonized with a given bacterial taxon if it had > 50% or ≤ 50% persistence, respectively, across the samples taken over the 3-year study period [3]. To visualise the distribution of chronic and intermittently colonizing bacteria taxa within each patient, the longitudinal persistence of every bacterial taxon observed in the lung microbiota over time was plotted against its mean relative abundance across the temporal samples it was detected in (Fig. 1). The resulting persistence-abundance relationships were all positive and significant. Wherein, the chronic infecting taxa were persistent and common, while the intermittent infecting taxa were typically rare and infrequent.

Mean taxa richness ± SD between patient cohorts was found to be significantly higher in the adult patients (Kruskal–Wallis: *H* = 8.55, *P* < 0.003). Where richness within the paediatric and adult groups was 147.8 ± 55.3 and 207.9 ± 49.4, respectively. No significant differences (*H* = 0.83, *P* = 0.361) in the mean richness of chronic colonizing taxa were observed between patient cohorts; paediatric taxa richness = 27.1 ± 8.6 and adult taxa richness = 24.1 ± 9.4. Conversely, the mean intermittent taxa richness was significantly higher (*H* = 9.29, *P* = 0.002) across adult patients (183.8 ± 42.5) when compared to paediatric patients (120 ± 49.4). While the intermittent taxa accounted for the majority of the microbiota diversity, the chronic taxa accounted for the majority of the relative abundance (chronic taxa mean abundance ± SD in the paediatric patients = 77.5% ± 10.1% and adult patients = 71.5% ± 11.4%).

### Temporal turnover and species-time relationships

**Fig. 2:**
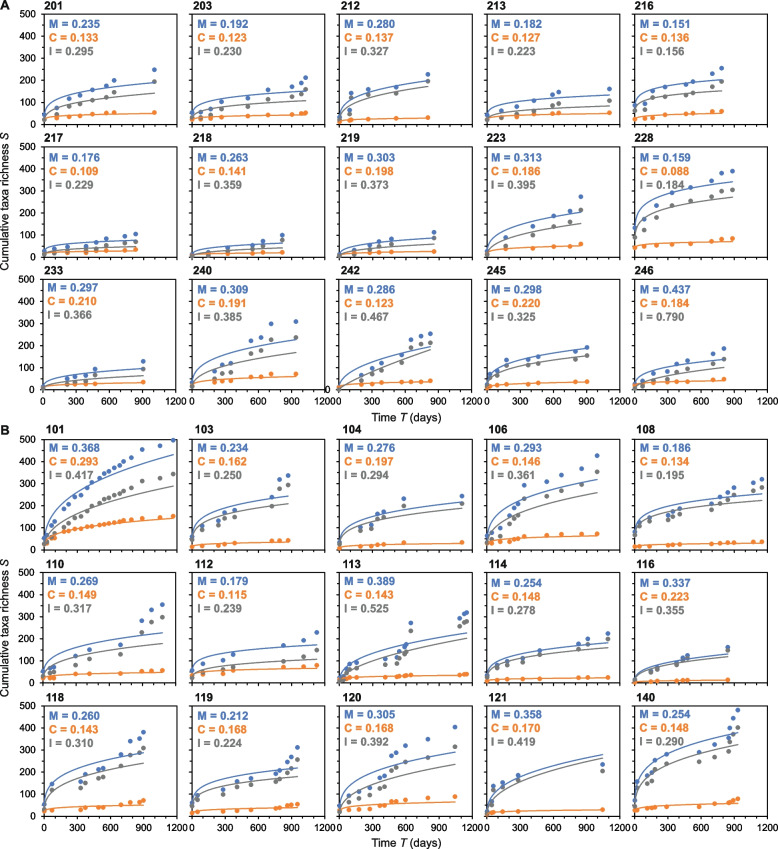
Species-time relationships for the lung microbiota within individual patients. Given for individual (**A**) paediatric and (**B**) adult patients are species-time relationships (STRs) for the microbiota (blue) and the chronic (orange) and intermittent (grey) colonizing taxa groups. Also given in each instance is the slope values (*w*) from the fitted STR models for the (M) microbiota and the (**C**) chronic and (I) intermittent colonizing taxa groups. All STRs were significant (*P* < 0.05 in all instances). Regression summary statistics are provided in Supplementary Table 2 and 3

Next, the contribution of the chronic and intermittent colonizing taxa on overall turnover within the wider lung microbiota was assessed. To measure turnover, we plotted for each patient the species-time relationships (STRs) for the chronic and intermittent colonizing taxa as well as the wider lung microbiota (Fig. 2). STRs describe how the observed species richness of a microbiota in a defined habitat increases with the length of time over which that microbiota is monitored [22, 25]. The STR is modelled with the power law equation *S* = *cT*^*w*^, where *S* is the number of observed species observed over time *T, c* is an empirically derived species- and patient-specific constant, and *w* is the slope of the fitted line or temporal scaling exponent [25]. Increasing values of *w* can be taken as greater values of temporal turnover [22].

The mean temporal scaling exponent for the lung microbiota across all patients was *w* = 0.286 ± 0.069, ranging from 0.151 to 0.437 (Fig. 2). Turnover was significantly higher for the intermittent taxa (mean *w* = 0.332 ± 0.120) than the chronic taxa (mean *w* = 0.161 ± 0.04)(*H* = 37.10, *P* < 0.0001). No significant differences in temporal turnover were observed between paediatric and adult patients for the whole microbiota (*H* = 0.31, *P* = 0.576), the chronic- (*H* = 1.30, *P* = 0.254), or the intermittent-colonizing taxa (*H* = 0.01, *P* = 0.950).

### Ecological process underpinning temporal turnover

**Fig. 3:**
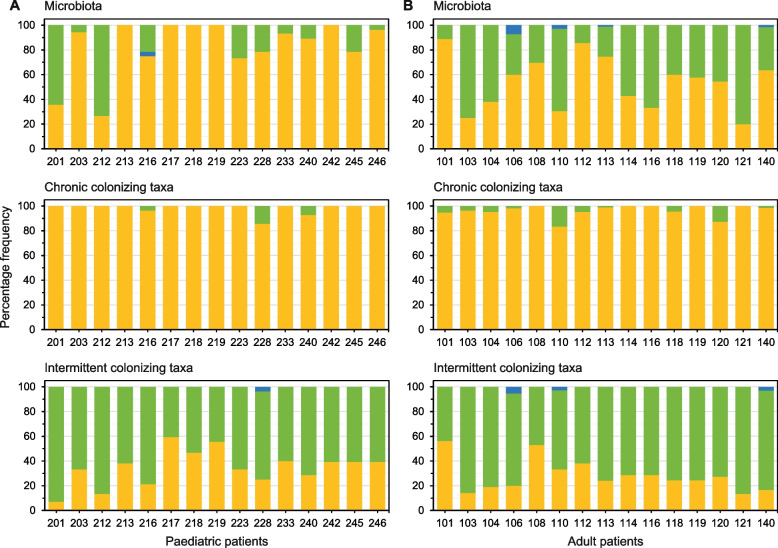
Ecological processes underpinning species turnover within patient lung microbiota. Given are percentage frequencies of Raup and Crick probability-based index pairwise values assigned to homogenizing dispersal (gold), drift (green), and dispersal limitation (blue) for the microbiota and the chronic and intermittent colonizing taxa groups within individual (**A**) paediatric and (**B**) adult patients

To assess the processes that underpin temporal turnover, we used Vellend’s simplified framework of ecological processes [40]. Within that framework, the ecological processes which could explain temporal turnover have been distilled into the influence and interplay between four rationalised processes; dispersal limitation, homogenizing dispersal, drift, and speciation [40, 42]. In brief, dispersal limitation results from biotic and abiotic pressures causing minimal exchange of organisms between microbiotas. Homogenizing dispersal is the degree to which individuals of species move between and successfully establish in local microbiota. Drift results from stochastic changes in population sizes, and speciation is the evolution of new species [22]. Here the Raup and Crick probability-based index of similarity (*S*_RC_) was used to test to what extent ecological processes (homogenizing dispersal, dispersal limitation, and drift) accounted for temporal turnover within chronic and intermittent colonizing taxa and the wider lung microbiota for each patient (Fig. 3).

Temporal turnover of chronic colonizing taxa was overwhelmingly characterized by the deterministic process of homogenizing dispersal in both the paediatric and adult patients (mean *S*_RC_ = 98.3 ± 3.6% and *S*_RC_ = 96.2 ± 4.7%, respectively), and to a lesser degree by drift (1.7 ± 3.9% and 3.8 ± 4.7%, respectively) (Fig. 3). Conversely, turnover of intermittent colonizing taxa was characterized by the stochastic process of drift for both paediatric and adult patients (mean *S*_RC_ = 65.1 ± 13.6% and *S*_RC_ = 71.1 ± 12.1%, respectively), followed by homogenizing dispersal (*S*_RC_ = 34.6 ± 13.7% and *S*_RC_ = 28.1 ± 12.3%, respectively), and slight influence from dispersal limitation (*S*_RC_ = 0.2 ± 0.9% and *S*_RC_ = 0.8 ± 1.6%) (Fig. 3).

Moreover, within the wider microbiota, the mean percentage frequency of homogenizing dispersal was significantly higher in paediatric patients (mean *S*_RC_ = 82.8 ± 22.4% [paediatric] versus *S*_RC_ = 53.7 ± 20.7% [adult]) (*H* = 10.93, *P* < 0.01) (Fig. 3). Whereas drift was significantly greater within the adult patients (mean *S*_RC_ = 17.0 ± 22.3% [paediatric] versus *S*_RC_ = 45.5 ± 20.9% [adult]) (*H* = 10.87, *P* < 0.01). Dispersal limitation had a negligible influence across all patients.

### Relationships between ecological processes and lung function

**Fig. 4:**
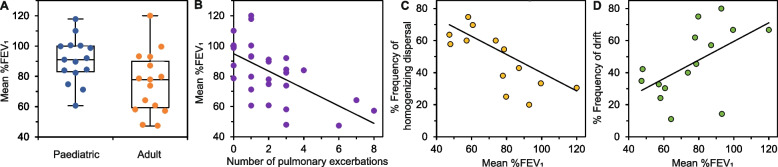
Relationships between lung function and ecological processes. (A) Comparison of mean lung function (%FEV_1_) over course of the study between paediatric and adult study patients; Kruskal–Wallis test *H* = 4.39, *P* = 0.03. **B** Relationship between lung function and number of acute pulmonary exacerbations experienced by each patient over course of study; *R*^2^ = 0.39, *F*_1,28_ = 17.4, *P* < 0.0001. **C**, **D** Relationships between lung function and ecological processes in adult patients; **C** *R*^2^ = 0.30, *F*_1,13_ = 5.44, *P* = 0.03 and (**D**) *R*^2^ = 0.31, *F*_1,13_ = 5.68, *P* = 0.03. Relationships for paediatric patients are not shown as both were nonsignificant. %FEV_1_ vs homogenizing dispersal: *R*^2^ = 0.001, *F*_1,13_ = 0.02, *P* = 0.865. %FEV_1_ vs drift: *R*.^2^ = 0.0003, *F*_1,13_ = 0.0001, *P* = 0.952

As could be expected in CF, lung function was significantly lower in adult patients (mean %FEV_1_ = 75.9 ± 18.9%) when compared to paediatric patients (90.5 ± 14.5%) and was inversely correlated with the number of acute pulmonary exacerbations experienced by patients over the course of the study (Fig. 4A, B) [1]. For the adult patients only, homogenizing dispersal and drift were found to significantly associate with lung function (Fig. 4C, D). Where a greater frequency of homogenizing dispersal was observed with worsening lung function. Conversely, the frequency of drift increased with better lung function.

## Discussion

Here we set out to understand the ecological patterns and processes of temporal turnover within the lung infection microbiota of paediatric and adult people with CF. Further, we aimed to ascertain the contribution of the constituent chronic and intermittent infecting bacteria on temporal turnover within the wider lung infection microbiota. The lung microbiota in individual patients were partitioned into chronic and intermittent species groups based on persistence using the clinically relevant Leeds criteria and visualized using persistence-abundance relationships (PARs) [4]. Strikingly, PARs across all paediatric and adult patients demonstrated that the commonness and rarity of bacterial species colonizing the airways of a patient is related to their temporal permanence (Fig. 1) [39]. In a clinical context, pathogen infection status in CF is based on culture-based presence-absence data alone and does not incorporate measures of pathogen abundance [4]. Moreover, culture has recently been found to significantly underestimate lung pathogen detection and chronic infection status in CF [3, 43, 44]. Here we found that bacterial taxa chronically infecting a patient were both persistent and common, whereas intermittent taxa were typically infrequent and rare. Or rather, the chronic and intermittent colonizing taxa respectively represent the resident and transient portions of the wider lung infection microbiota. Similar relationships have been previously observed in a spatial context, in the form of occupancy-abundance relationships for core and satellite species distributions in cross-sectional studies, e.g., [7, 11, 45]. However, this is the first time this has been applied to taxa distributions within patient microbiota through time.

PARs represent a novel means of visualising the temporal distributions of individual bacterial taxa that chronically and intermittently infect the airways of a patient. They highlight that patients can be chronically colonized with multiple bacterial species, including canonical CF pathogens. Moreover, our data demonstrates it is possible for a patient to be chronically and/or intermittently colonized with multiple canonical CF pathogens (Fig. 1). Our data clearly indicates that the true extent of chronic infection in a patient is missed when applying traditional culture-based ‘one microbe, one disease’ approaches of infection pathogenesis to CF [3, 7]. More broadly, this further highlights an inadequacy in traditional models of lung infection and a need to move to therapeutic targeting of the lung microbiota in general [14, 16, 46].

The lungs can be considered as ecological island habitats that are open to immigration of bacteria from the upper airways, oral cavity, and wider environment, with those same bacteria also subject to elimination and extinction resulting from, for example, mucociliary clearance, host immune responses, and antimicrobial interventions [7, 13, 21]. To measure turnover, we plotted STRs constructed with an approach that accounts for both immigration and extinction within a microbiota through time (Fig. 2) [22]. All temporal turnover (*w*) values were within the ranges observed for STRs from a wide range of microbial, animal, and plant communities [47, 48]. We also found temporal turnover was significantly higher in the intermittent taxa when compared to the chronic taxa. This indicated that the intermittent colonizing taxa primarily drives turnover of the wider lung microbiota, while turnover in the chronic taxa is conserved in comparison. From an ecological perspective, this would seem logical as intermittent colonizing taxa account for the transient species and newly arrived immigrants, which have a higher probability of going locally extinct due to smaller population sizes [19, 39]. Conversely, the resident chronic colonizing taxa are invariably common with larger established species populations (Figs. 1 and 2) which are less likely to become locally extinct in comparison and as such would be more difficult to eradicate [19, 39]. This therefore adds newfound support to the established approach of early eradication of recently acquired infection to prevent the transition to chronic infection.

From an island biogeography perspective, the lungs of children and adults could be considered as small versus large island systems, respectively. As such, theoretical predictions from island biogeography anticipate that smaller islands have lower species richness and greater turnover, whereas larger islands have higher richness and reduced turnover over time; for example, due to less available physical niche space and habitat heterogeneity in the former and more in the latter [19, 22, 24]. Although significantly lower microbiota richness was observed in paediatric patients, no significant differences in turnover were observed between paediatric and adult patients. We posit there may exist a lack of direct equivalence in lung habitat similarity between children and adult patients with CF, primarily resulting from the cumulative effects of CF pulmonary disease experienced with increasing age along with the spectrum of disease severity dictated by the CFTR mutations that individual patients have inherited [1]. Further, CF lung microbiota are highly personalized to the individual patient [7], and it would also appear that the degree of temporal variability, as observed here within the STRs, is also highly personalized [27].

There is a recognised need to understand the ecological processes and mechanisms that underpin spatial and temporal patterns of species distribution and turnover [17, 22, 42]. Temporal turnover of the chronic taxa was found to be predominantly driven by the deterministic process of homogenizing dispersal (Fig. 3). This could be expected given that chronic taxa are composed of species which are temporally persistent with large population sizes. Conversely, the turnover of intermittent colonizing taxa was characterized by the stochastic process of drift, followed by homogenizing dispersal (Fig. 3). The higher levels of drift within the intermittent taxa are likely due to the transient species having an increased probability of experiencing chance events [19]. Where stochastic events include death, reproduction, and migration, and all underpin the process of drift [22, 41]. Moreover, a substantial degree of homogenizing dispersal could be anticipated from those species transitioning from transient intermittent status to established chronic infection status over time [49]. We therefore posit that in the wider microbiota, the influence of homogenizing dispersal and drift is largely due to the distinct ecological properties of the constituent chronic and intermittent taxa. Interestingly, the observation within the wider microbiota of higher homogenizing dispersal in paediatric patients and greater drift in adult patients is intriguing as the opposite could be anticipated, i.e., a shift from the stochastic process of drift with increasing age to greater frequencies of the deterministic process of homogenizing dispersal, attributable to progressive airway and lung parenchymal damage resulting from a vicious cycle of unchecked airway infection and inflammation [7, 50].

With regards to speciation, this process is not directly accounted for using the *S*_RC_ index, nonetheless, it can cause differences in diversity among sets of communities that do not exchange individuals through dispersal [22, 40]. Consequently, speciation should have negligible influence within a set of communities where individuals disperse among local communities within a spatial or temporal metacommunity [22, 51]. This was the case in the current study where dispersal limitation either had negligible or no influence. Furthermore, speciation should be negligible given the longitudinal timeframe of this study and the method used to define taxa, i.e., 16S rRNA gene amplicon sequencing [22].

The observed relationships between homogenizing dispersal and drift with lung function in only adult patients could well be a response to increasing selection pressure with reducing lung function and associated worse clinical outcomes [7] (Fig. 4). Similar has been observed for bacterial communities in model systems when a specific selective pressure was experimentally increased [25]. An explanation for why this was observed only in adult patients could be, again, attributable to more progressive airway and lung parenchymal damage experienced with increasing age. It could also be the case that the paediatric patients within the study had, along with better lung function, less range and variation in %FEV_1_ compared to the adult patients. Therefore, studies with larger patient numbers and hence the potential for more variation could elucidate whether that was a factor or not.

There are potential limitations to this study that deserve consideration. Both sputum and cough swabs (taken when a patient was not sputum-productive) samples were taken across the study and this could have introduced bias to the underlying microbiota characteristics. We found that the number of sequence reads per sample was significantly higher in sputum samples, but taxa richness and compositional similarities were not significantly different between sample types (Supplementary Table S4). Further, the maximum sampling duration and number of samples per patient were not uniform within the study. However, we found that richness and STR scaling exponents were not significantly affected by either potential factor (Supplementary Table 5 and 6). Finally, respiratory samples were collected before the widespread availability of effective CF transmembrane conductance regulator (CFTR) modulators. It is not known how the lung infection microbiota will be affected longitudinally in CFTR-modulated paediatric and adult patients. This will be a subject for our future work. Importantly, the current study establishes an invaluable pre-CFTR modulator therapy baseline to compare to in such future work.

## Conclusions

Lower airway infections across a range of chronic respiratory diseases are comprised of diverse and temporally dynamic lung microbiota. In diseases like CF where the primary cause of morbidity and early mortality is chronic lung infection, understanding the dynamics of microbiota turnover and the contribution of the chronic and intermittent infection elements is crucial. Set within an ecological framework and drawing upon concepts central to the theory of island biogeography, we determined the patterns and processes underpinning temporal turnover within the dynamic lung microbiota of individual paediatric and adult CF patients. We establish that in all patients, chronically infecting taxa represent the common and conserved resident portion of the lung microbiota, underpinned principally by the deterministic process of homogenizing dispersal. Conversely, intermittent colonizing taxa drive turnover in the wider microbiota, as they account for the rarer and highly dynamic transient species along with newly arrived immigrants and are predominantly driven by the stochastic process of drift. Our findings add newfound support to the established approach of early eradication of recently acquired infection to prevent the transition to chronic infection. Further, our findings clearly indicate the extent of chronic colonization in individual patients is far greater than is appreciated through clinical microbiological culture alone. Combined, this study further illustrates the inadequacy of traditional ‘one microbe, one disease’ models of lung microbiology and infection pathogenesis. While culture has been useful for clinical microbiological surveillance, it has repeatedly been shown to be both limited and biased in CF, e.g., [3, 43, 44]. Given the unquestionable polymicrobial nature of CF lung infection, it is sensible to recommend using molecular approaches that can define all microbial species within a patient’s lung infection microbiota. Moreover, when combined with a novel ecological framework for understanding the temporal dynamics of polymicrobial infection in CF has translational potential to guide and improve therapeutic targeting of lung microbiota in CF and across a range of chronic airway diseases.

### Supplementary Information

**Additional file 1: Supplementary Table S1.** Persistence-abundance regression statistics from lung microbiota from paediatric and adult patients.

**Additional file 2: Supplementary Table S2.** Species-time relationship regression statistics for the lung microbiota, the chronic- and, intermittent- colonizing taxa from the paediatric patients.

**Additional file 3: Supplementary Table S3.** Species-time relationship regression statistics for the lung microbiota, the chronic- and, intermittent- colonizing taxa from the adult patients.

**Additional file 4: Supplementary Table S4.** Comparisons of microbiota characteristics between sputum and cough swab samples. (A) and (B) Comparisons of taxa richness and number of sequence reads between sample types using Kruskal–Wallis tests. Indicated are number of samples (N) in each group, mean values and standard deviation (SD), and Kruskal–Wallis test statistic (*H*) and significance (*P*). (C) Comparison of Bray–Curtis indices of compositional similarity between samples groups. Given are number of samples (*N*), number of pairwise comparisons (*n*), mean values and SD, and Analysis of similarities (ANOSIM) test statistic (*R*) and significance (*P*).

**Additional file 5: Supplementary Table S5.** Relationships between total taxa richness across samples from individual patients and maximum sampling duration (days) or number of samples in adult and paediatric patients. Given are regression summary statistics: Coefficient of determination (*R*^2^), *F*-statistic, and significance (*P*). Degrees of freedom were 1,13 in all instances.

**Additional file 6: Supplementary Table S6.** Relationships between slope values (*w*) from species-time relationships and maximum sampling duration (days) or number of samples in adult and paediatric patients. Given are regression summary statistics: Coefficient of determination (*R*^2^), *F*-statistic, and significance (*P*). Degrees of freedom were 1,13 in all instances.

## Data Availability

The raw sequence data reported in this study have been deposited in the European Nucleotide Archive under study accession number PRJEB62148. Anonymised clinical metadata and processed microbiota data have been deposited at figshare.com under https://figshare.com/s/f7c35c6e89448c4a3aa1
