# Supplementary material for: Ecological patterns and processes of temporal turnover within lung infection microbiota
Source: Microbiome. 2024 Mar 25;12:63. doi: 10.1186/s40168-024-01780-6 (PMC10962200; doi:10.1186/s40168-024-01780-6)
Supplement: Supplementary file 2 — Additional file 1: Supplementary Table S1. Persistence-abundance regression statistics from lung microbiota from paediatric and adult patients. [file 40168_2024_1780_MOESM1_ESM.docx]

**Supplementary Table 1** Persistence-abundance regression statistics from lung microbiota from paediatric and adult patients.

|  | **Patient** | ***R*^2^** | **df** | ***F*** | ***P*** |
| --- | --- | --- | --- | --- | --- |
| Paediatric | 201 | 0.832 | 1,162 | 363.6 | <0.0001 |
|  | 203 | 0.851 | 1,127 | 334.9 | <0.0001 |
|  | 212 | 0.728 | 1,175 | 197.5 | <0.0001 |
|  | 213 | 0.745 | 1,107 | 133.8 | <0.0001 |
|  | 216 | 0.787 | 1,175 | 285.2 | <0.0001 |
|  | 217 | 0.856 | 1,67 | 183.1 | <0.0001 |
|  | 218 | 0.785 | 1,76 | 121.8 | <0.0001 |
|  | 219 | 0.788 | 1,86 | 141.2 | <0.0001 |
|  | 223 | 0.776 | 1,197 | 298.2 | <0.0001 |
|  | 228 | 0.814 | 1,276 | 542.8 | <0.0001 |
|  | 233 | 0.743 | 1,101 | 124.6 | <0.0001 |
|  | 240 | 0.815 | 1,196 | 386.8 | <0.0001 |
|  | 242 | 0.806 | 1,132 | 245.1 | <0.0001 |
|  | 245 | 0.824 | 1,132 | 279.2 | <0.0001 |
|  | 246 | 0.846 | 1,187 | 471.8 | <0.0001 |
| Adults | 101 | 0.842 | 1,205 | 500.7 | <0.0001 |
|  | 103 | 0.622 | 1,235 | 148.3 | <0.0001 |
|  | 104 | 0.663 | 1,163 | 128.1 | <0.0001 |
|  | 106 | 0.750 | 1,283 | 363.6 | <0.0001 |
|  | 108 | 0.822 | 1,199 | 414.00 | <0.0001 |
|  | 110 | 0.755 | 1,249 | 329.5 | <0.0001 |
|  | 112 | 0.802 | 1,149 | 268.3 | <0.0001 |
|  | 113 | 0.838 | 1,209 | 492.8 | <0.0001 |
|  | 114 | 0.693 | 1,159 | 146.6 | <0.0001 |
|  | 116 | 0.690 | 1,121 | 109.8 | <0.0001 |
|  | 118 | 0.791 | 1,236 | 393.5 | <0.0001 |
|  | 119 | 0.725 | 1,200 | 221.6 | <0.0001 |
|  | 120 | 0.771 | 1,231 | 338.3 | <0.0001 |
|  | 121 | 0.640 | 1,178 | 123.7 | <0.0001 |
|  | 140 | 0.787 | 1,286 | 464.6 | <0.0001 |

df denotes degrees of freedom.
