# Supplementary material for: Ecological patterns and processes of temporal turnover within lung infection microbiota
Source: Microbiome. 2024 Mar 25;12:63. doi: 10.1186/s40168-024-01780-6 (PMC10962200; doi:10.1186/s40168-024-01780-6)
Supplement: Supplementary file 3 — Additional file 2: Supplementary Table S2. Species-time relationship regression statistics for the lung microbiota, the chronic- and, intermittent- colonizing taxa from the paediatric patients. [file 40168_2024_1780_MOESM2_ESM.docx]

**Supplementary Table 2** Species-time relationship regression statistics for the lung microbiota, the chronic- and, intermittent- colonizing taxa from the paediatric patients.

| **Patient** |  | ***w*** | ***c*** | ***R*^2^** | **df** | ***F*** | ***P*** |
| --- | --- | --- | --- | --- | --- | --- | --- |
| 201 | Microbiota | 0.2349 | 1.574 | 0.85 | 1,6 | 34.51 | 0.001 |
|  | Chronic | 0.1330 | 1.298 | 0.80 | 1,6 | 24.16 | 0.003 |
|  | Intermittent | 0.2954 | 1.266 | 0.88 | 1,6 | 43.60 | 0.001 |
| 203 | Microbiota | 0.1924 | 1.595 | 0.66 | 1,7 | 13.73 | 0.008 |
|  | Chronic | 0.1230 | 1.263 | 0.70 | 1,7 | 16.51 | 0.005 |
|  | Intermittent | 0.2302 | 1.339 | 0.65 | 1,7 | 13.21 | 0.008 |
| 212 | Microbiota | 0.2803 | 1.483 | 0.87 | 1,4 | 26.28 | 0.007 |
|  | Chronic | 0.1372 | 1.054 | 0.93 | 1,4 | 56.00 | 0.002 |
|  | Intermittent | 0.3267 | 1.287 | 0.87 | 1,4 | 25.56 | 0.007 |
| 213 | Microbiota | 0.1817 | 1.572 | 0.79 | 1,5 | 18.22 | 0.008 |
|  | Chronic | 0.1270 | 1.307 | 0.82 | 1,5 | 22.14 | 0.005 |
|  | Intermittent | 0.2231 | 1.244 | 0.77 | 1,5 | 17.12 | 0.009 |
| 216 | Microbiota | 0.1505 | 1.871 | 0.72 | 1,6 | 15.16 | 0.008 |
|  | Chronic | 0.1356 | 1.308 | 0.68 | 1,6 | 12.55 | 0.012 |
|  | Intermittent | 0.1558 | 1.731 | 0.70 | 1,6 | 14.20 | 0.009 |
| 217 | Microbiota | 0.1761 | 1.371 | 0.70 | 1,6 | 14.02 | 0.01 |
|  | Chronic | 0.1092 | 1.135 | 0.67 | 1,6 | 12.18 | 0.013 |
|  | Intermittent | 0.2293 | 1.012 | 0.71 | 1,6 | 14.86 | 0.008 |
| 218 | Microbiota | 0.2629 | 1.039 | 0.77 | 1,4 | 13.22 | 0.022 |
|  | Chronic | 0.1410 | 0.886 | 0.92 | 1,4 | 43.35 | 0.003 |
|  | Intermittent | 0.3593 | 0.580 | 0.72 | 1,4 | 10.16 | 0.033 |
| 219 | Microbiota | 0.3005 | 1.060 | 0.9 | 1,4 | 35.86 | 0.004 |
|  | Chronic | 0.1950 | 0.847 | 0.99 | 1,4 | 955.27 | <0.0001 |
|  | Intermittent | 0.3707 | 0.687 | 0.81 | 1,4 | 17.50 | 0.014 |
| 223 | Microbiota | 0.3132 | 1.392 | 0.94 | 1,4 | 57.84 | 0.002 |
|  | Chronic | 0.1855 | 1.169 | 0.97 | 1,4 | 119.25 | <0.0001 |
|  | Intermittent | 0.3949 | 1.027 | 0.93 | 1,4 | 56.62 | 0.002 |
| 228 | Microbiota | 0.1589 | 2.065 | 0.84 | 1,6 | 31.09 | 0.001 |
|  | Chronic | 0.0880 | 1.589 | 0.67 | 1,6 | 11.97 | 0.013 |
|  | Intermittent | 0.1840 | 1.892 | 0.86 | 1,6 | 35.70 | 0.001 |
| 233 | Microbiota | 0.3123 | 1.392 | 0.94 | 1,4 | 57.84 | 0.002 |
|  | Chronic | 0.1855 | 1.169 | 0.97 | 1,4 | 119.25 | <0.0001 |
|  | Intermittent | 0.3949 | 1.027 | 0.93 | 1,4 | 56.62 | 0.002 |
| 240 | Microbiota | 0.3087 | 1.438 | 0.81 | 1,6 | 24.95 | 0.002 |
|  | Chronic | 0.1910 | 1.210 | 0.83 | 1,6 | 29.24 | 0.002 |
|  | Intermittent | 0.3846 | 1.083 | 0.83 | 1,6 | 28.93 | 0.002 |
| 242 | Microbiota | 0.2862 | 1.306 | 0.83 | 1,6 | 28.30 | 0.002 |
|  | Chronic | 0.1230 | 1.255 | 0.82 | 1,6 | 27.77 | 0.002 |
|  | Intermittent | 0.4673 | 0.647 | 0.89 | 1,6 | 49.60 | <0.0001 |
| 245 | Microbiota | 0.2978 | 1.394 | 0.99 | 1,6 | 414.87 | <0.0001 |
|  | Chronic | 0.2204 | 0.894 | 0.98 | 1,6 | 378.04 | <0.0001 |
|  | Intermittent | 0.3254 | 1.228 | 0.98 | 1,6 | 304.10 | <0.0001 |
| 246 | Microbiota | 0.4366 | 1.017 | 0.93 | 1,6 | 73.27 | <0.0001 |
|  | Chronic | 0.1842 | 1.022 | 0.95 | 1,6 | 105.12 | <0.0001 |
|  | Intermittent | 0.7897 | 0.045 | 0.99 | 1,6 | 417.21 | <0.0001 |

Given are w and c which are the slope and intercept from each STR. ‘df’ denotes degrees of freedom.
