# Supplementary material for: Ecological patterns and processes of temporal turnover within lung infection microbiota
Source: Microbiome. 2024 Mar 25;12:63. doi: 10.1186/s40168-024-01780-6 (PMC10962200; doi:10.1186/s40168-024-01780-6)
Supplement: Supplementary file 4 — Additional file 3: Supplementary Table S3. Species-time relationship regression statistics for the lung microbiota, the chronic- and, intermittent- colonizing taxa from the adult patients. [file 40168_2024_1780_MOESM3_ESM.docx]

**Supplementary Table 3** Species-time relationship regression statistics for the lung microbiota, the chronic- and, intermittent- colonizing taxa from the adult patients.

| **Patient** |  | ***w*** | ***c*** | ***R*^2^** | **df** | ***F*** | ***P*** |
| --- | --- | --- | --- | --- | --- | --- | --- |
| 101 | Microbiota | 0.3676 | 1.507 | 0.90 | 1,18 | 155.23 | <0.0001 |
|  | Chronic | 0.2931 | 1.257 | 0.91 | 1,18 | 181.61 | <0.0001 |
|  | Intermittent | 0.4165 | 1.184 | 0.89 | 1,18 | 142.02 | <0.0001 |
| 103 | Microbiota | 0.2336 | 1.700 | 0.82 | 1,6 | 26.99 | 0.002 |
|  | Chronic | 0.1618 | 1.069 | 0.71 | 1,6 | 14.52 | 0.009 |
|  | Intermittent | 0.2498 | 1.586 | 0.83 | 1,6 | 30.04 | 0.002 |
| 104 | Microbiota | 0.2760 | 1.496 | 0.92 | 1,5 | 54.47 | 0.001 |
|  | Chronic | 0.1973 | 0.865 | 0.85 | 1,5 | 27.20 | 0.003 |
|  | Intermittent | 0.2939 | 1.380 | 0.93 | 1,5 | 63.08 | 0.001 |
| 106 | Microbiota | 0.2930 | 1.625 | 0.76 | 1,9 | 28.85 | <0.0001 |
|  | Chronic | 0.1460 | 1.366 | 0.67 | 1,9 | 17.99 | 0.002 |
|  | Intermittent | 0.3608 | 1.332 | 0.80 | 1,9 | 34.82 | <0.0001 |
| 108 | Microbiota | 0.1856 | 1.836 | 0.84 | 1,10 | 52.76 | <0.0001 |
|  | Chronic | 0.1344 | 1.069 | 0.68 | 1,10 | 21.06 | 0.001 |
|  | Intermittent | 0.1946 | 1.754 | 0.86 | 1,10 | 59.40 | <0.0001 |
| 110 | Microbiota | 0.2693 | 1.539 | 0.71 | 1,7 | 16.77 | 0.005 |
|  | Chronic | 0.1492 | 1.225 | 0.66 | 1,7 | 13.83 | 0.007 |
|  | Intermittent | 0.3172 | 1.290 | 0.73 | 1,7 | 18.63 | 0.003 |
| 112 | Microbiota | 0.1788 | 1.691 | 0.82 | 1,5 | 22.21 | 0.005 |
|  | Chronic | 0.1152 | 1.470 | 0.67 | 1,5 | 10.34 | 0.024 |
|  | Intermittent | 0.2393 | 1.300 | 0.89 | 1,5 | 39.45 | 0.002 |
| 113 | Microbiota | 0.3889 | 1.165 | 0.83 | 1,12 | 58.56 | <0.0001 |
|  | Chronic | 0.1430 | 1.103 | 0.79 | 1,12 | 45.51 | <0.0001 |
|  | Intermittent | 0.5252 | 0.699 | 0.88 | 1,12 | 88.22 | <0.0001 |
| 114 | Microbiota | 0.2544 | 1.487 | 0.93 | 1,6 | 83.14 | <0.0001 |
|  | Chronic | 0.1476 | 0.913 | 0.90 | 1,6 | 54.98 | <0.0001 |
|  | Intermittent | 0.2783 | 1.358 | 0.94 | 1,6 | 93.17 | <0.0001 |
| 116 | Microbiota | 0.3367 | 1.133 | 0.95 | 1,5 | 89.08 | <0.0001 |
|  | Chronic | 0.2258 | 0.442 | 0.90 | 1,5 | 45.46 | 0.001 |
|  | Intermittent | 0.3554 | 1.036 | 0.95 | 1,5 | 99.31 | <0.0001 |
| 118 | Microbiota | 0.2604 | 1.690 | 0.88 | 1,8 | 56.51 | <0.0001 |
|  | Chronic | 0.1434 | 1.279 | 0.58 | 1,8 | 10.92 | 0.011 |
|  | Intermittent | 0.3099 | 1.466 | 0.92 | 1,8 | 91.29 | <0.0001 |
| 119 | Microbiota | 0.2118 | 1.709 | 0.82 | 1,8 | 0.82 | <0.0001 |
|  | Chronic | 0.1676 | 1.093 | 0.66 | 1,8 | 15.41 | 0.004 |
|  | Intermittent | 0.2235 | 1.588 | 0.84 | 1,8 | 42.32 | <0.0001 |
| 120 | Microbiota | 0.3045 | 1.544 | 0.78 | 1,9 | 32.38 | <0.0001 |
|  | Chronic | 0.1681 | 1.302 | 0.53 | 1,9 | 9.98 | 0.012 |
|  | Intermittent | 0.3923 | 1.187 | 0.88 | 1,9 | 63.15 | <0.0001 |
| 121 | Microbiota | 0.3579 | 1.365 | 0.94 | 1,4 | 60.02 | 0.001 |
|  | Chronic | 0.1701 | 0.947 | 0.99 | 1,4 | 440.66 | <0.0001 |
|  | Intermittent | 0.4191 | 1.155 | 0.93 | 1,4 | 52.88 | 0.002 |
| 140 | Microbiota | 0.2539 | 1.824 | 0.93 | 1,10 | 141.03 | <0.0001 |
|  | Chronic | 0.1478 | 1.324 | 0.72 | 1,10 | 25.80 | <0.0001 |
|  | Intermittent | 0.2899 | 1.650 | 0.95 | 1,10 | 184.81 | <0.0001 |

Given are w and c which are the slope and intercept from each STR. ‘df’ denotes degrees of freedom.
