# Supplementary material for: Ecological patterns and processes of temporal turnover within lung infection microbiota
Source: Microbiome. 2024 Mar 25;12:63. doi: 10.1186/s40168-024-01780-6 (PMC10962200; doi:10.1186/s40168-024-01780-6)
Supplement: Supplementary file 5 — Additional file 4: Supplementary Table S4. Comparisons of microbiota characteristics between sputum and cough swab samples. (A) and (B) Comparisons of taxa richness and number of sequence reads between sample types using Kruskal–Wallis tests. Indicated are number of samples (N) in each group, mean values and standard deviation (SD), and Kruskal–Wallis test statistic (H) and significance (P). (C) Comparison of Bray–Curtis indices of compositional similarity between samples groups. Given are number of samples (N), number of pairwise comparisons (n), mean values and SD, and Analysis of similarities (ANOSIM) test statistic (R) and significance (P). [file 40168_2024_1780_MOESM4_ESM.docx]

**Supplementary Table 4** Comparisons of microbiota characteristics between sputum and cough swab samples. (A) and (B) Comparisons of taxa richness and number of sequence reads between sample types using Kruskal-Wallis tests. Indicated are number of samples (N) in each group, mean values and standard deviation (SD), and Kruskal-Wallis test statistic (*H*) and significance (*P*). (C) Comparison of Bray-Curtis indices of compositional similarity between samples groups. Given are number of samples (*N*), number of pairwise comparisons (*n*), mean values and SD, and Analysis of similarities (ANOSIM) test statistic (*R*) and significance (*P*).

| **A** |  | |  | |  |  | |  | |  | |
| --- | --- | --- | --- | --- | --- | --- | --- | --- | --- | --- | --- |
|  | *N* | |  | | Mean richness | SD | | *H* | | *P* | |
| Sputum samples | 190 | |  | | 50.6 | 21.4 | | 2.52 | | 0.112 | |
| Cough swab samples | 72 | |  | | 48.4 | 30.4 | |  | |  | |
|  |  | |  | |  |  | |  | |  | |
| **B** |  | |  | |  |  | |  | |  | |
|  | *N* | |  | | Mean sequence reads | SD | | *H* | | *P* | |
| Sputum samples | 190 | |  | | 20955.8 | 11635.5 | | 14.04 | | <0.0001 | |
| Cough swab samples | 72 | |  | | 15465.4 | 10220.1 | |  | |  | |
|  |  | |  | |  |  | |  | |  | |
| **C** |  | |  | |  |  | |  | |  | |
|  | *N* | *n* | | Mean similarity | | SD | *R* | | *P* | |  |
| Between groups | 262 | | 13680 | | 0.31 | 0.15 | 0.018 | | | 0.271 | |
| Within sputum samples | 190 | | 17955 | | 0.29 | 0.12 |  | | |  | |
| Within cough swab samples | 72 | | 2556 | | 0.32 | 0.13 |  | | |  | |
|  |  | |  | |  |  | |  | |  | |
