# Supplementary material for: Ecological patterns and processes of temporal turnover within lung infection microbiota
Source: Microbiome. 2024 Mar 25;12:63. doi: 10.1186/s40168-024-01780-6 (PMC10962200; doi:10.1186/s40168-024-01780-6)
Supplement: Supplementary file 6 — Additional file 5: Supplementary Table S5. Relationships between total taxa richness across samples from individual patients and maximum sampling duration (days) or number of samples in adult and paediatric patients. Given are regression summary statistics: Coefficient of determination (R2), F-statistic, and significance (P). Degrees of freedom were 1,13 in all instances. [file 40168_2024_1780_MOESM5_ESM.docx]

**Supplementary Table 5** Relationships between total taxa richness across samples from individual patients and maximum sampling duration (days) or number of samples in adult and paediatric patients. Given are regression summary statistics: Coefficient of determination (*R*^2^), *F*-statistic, and significance (*P*). Degrees of freedom were 1,13 in all instances.

| Patients | Predictor | Taxa richness | *R*^2^ | *F* | *P* |
| --- | --- | --- | --- | --- | --- |
| Adults | Maximum duration (Days) | Microbiota | 0.04 | 0.50 | 0.490 |
|  |  | Chronic | 0.10 | 1.44 | 0.251 |
|  |  | Intermittent | 0.08 | 1.08 | 0.391 |
|  | Number of samples | Microbiota | 0.16 | 2.40 | 0.145 |
|  |  | Chronic | 0.26 | 4.69 | 0.06 |
|  |  | Intermittent | 0.09 | 1.35 | 0.267 |
| Paediatrics | Maximum duration (Days) | Microbiota | 0.005 | 0.07 | 0.799 |
|  |  | Chronic | 0.04 | 0.55 | 0.470 |
|  |  | Intermittent | 0.01 | 0.18 | 0.682 |
|  | Number of samples | Microbiota | 0.07 | 0.90 | 0.359 |
|  |  | Chronic | 0.04 | 0.51 | 0.488 |
|  |  | Intermittent | 0.06 | 0.88 | 0.366 |
