# Supplementary material for: Ecological patterns and processes of temporal turnover within lung infection microbiota
Source: Microbiome. 2024 Mar 25;12:63. doi: 10.1186/s40168-024-01780-6 (PMC10962200; doi:10.1186/s40168-024-01780-6)
Supplement: Supplementary file 7 — Additional file 6: Supplementary Table S6. Relationships between slope values (w) from species-time relationships and maximum sampling duration (days) or number of samples in adult and paediatric patients. Given are regression summary statistics: Coefficient of determination (R2), F-statistic, and significance (P). Degrees of freedom were 1,13 in all instances. [file 40168_2024_1780_MOESM6_ESM.docx]

**Supplementary Table 6** Relationships between slope values (*w*) from species-time relationships and maximum sampling duration (days) or number of samples in adult and paediatric patients. Given are regression summary statistics: Coefficient of determination (*R*^2^), *F*-statistic, and significance (*P*). Degrees of freedom were 1,13 in all instances.

| Patients | Predictor | *w* | *R*^2^ | *F* | *P* |
| --- | --- | --- | --- | --- | --- |
| Adults | Maximum duration (days) | Microbiota | 0.01 | 0.12 | 0.732 |
|  |  | Chronic taxa | 0.0001 | 0.001 | 0.955 |
|  |  | Intermittent taxa | 0.13 | 1.91 | 0.190 |
|  | Number of samples | Microbiota | 0.12 | 1.75 | 0.208 |
|  |  | Chronic taxa | 0.20 | 3.33 | 0.091 |
|  |  | Intermittent taxa | 0.003 | 0.03 | 0.860 |
| Paediatrics | Maximum duration (days) | Microbiota | 0.12 | 1.75 | 0.333 |
|  |  | Chronic taxa | 0.2 | 3.33 | 0.835 |
|  |  | Intermittent taxa | 0.003 | 0.03 | 0.293 |
|  | Number of samples | Microbiota | 0.08 | 1.10 | 0.313 |
|  |  | Chronic taxa | 0.13 | 1.86 | 0.196 |
|  |  | Intermittent taxa | 0.01 | 0.14 | 0.716 |
